# Books and Magazines

**Published:** 1907-07

**Authors:** 


### Books and Magazines.

Diseases of Children, A Manual of. By W. F. Radue, M. D., Chicago. The Clinic Pub. Co., 1907. Cloth. Pp., 165. Price, $1.00.

The teachings of this little book are drawn from the personal experiences of the author with the active principles and other latter-day medicaments. It will be of unusual interest to progressive physicians, as the tendency now is, and very properly, to cut out the prescription druggist, who is apt to dispense "something just as good,"—and to dispense one's own medicine. This can and should be done; the alkaloid and other active principles can be carried in a neat case, and your patient saved an exorbitant drug bill when he gets well—or the estate—if he doesn't. Buy Dr. Radue's book and read it. You will find it "worth while."

Diseases of the Rectum and Intestines, Their Consequences and Non-Surgical Treatment. By W. C. Brinkerhoff, M. D., Chicago. Illustrated. Orban Pub. Co., Chicago, 1907. Pp., 158. Cloth, $2.00.

This volume embodies the views of the author arrived at by a large experience in the treatment of certain very troublesome diseases without operation; and we gather from a brief examination of its pages that those views are at variance with those held by most writers. So much the more reason for reading it. You can not learn from those who agree with you in everything.

---

The Standard Family Physician. Smith Ely Jelliffe, *et alia*. Two volumes. Large 8vo; in buckram binding; $15 per set, net; in three-quarters Morocco, $20, net. Illustrated. Funk & Wagnalls Company, 1907.

In a recent lecture, Dr. Osler, enunciating the principle that the physician was the teacher rather than the servant of the patient, declared that in the matter of drugs, his therapeutic practice at Johns Hopkins University has been wittily summed up as a mixture of nux vomica and hope. He declared that "Medicine as it is used to day is all moonshine and there are not many drugs that have a therapeutic value." In an age like our own, in which the drug habit has taken firm hold on the people, it would be well if some attention were paid to his remarks on the uselessness of most drugs. But will the people listen? The trouble with most people is that they are to much the subject of exploitation with patent medicines to pay much attention to his advice. It is hoped, however, that a book of the character of the Standard Family Physician, the avowed purpose of which is to check the harm done by patent medicines and the many pseudo-medical treatises foisted on the public, will meet with a ready demand. It aims to supplant by an authoritative and popular work, all second-rate books on household medicine and all spurious works—works devoted to the exploitation of quack medicine and its attendant evils.

The present treatise is the result of the joint collaboration of Dr. Carl Reissig, of Hamburg, assisted by an able corps of European specialists in medicine and surgery, and Dr. Smith Ely Jelliffe, associate editor of the *New York Medical Journal,* in conjunction with a number of other eminent American physicians. This work has been written in such a way that it can be easily understood by any intelligent layman, no matter what subjects it treats. Its range is exhaustive, and it is especially valuable in its anatomical and clinical parts. The arrangement commends itself for rapid consultation. The first section is devoted to the presentation and study of the anatomy of the human body; this is accompanied by a manakin (in colors) which shows its 345 distinct parts.

The section contains articles explaining the structure, development, and functions of the human skin, the bones, joints, ligaments, muscles, and internal organs. Much space is given to explanations of the circulatory system and nervous system, and the function of the organs of special sense and of generation are also treated.

The second section of the Standard Family Physician is devoted exclusively to the description of diseases, their methods of treatment, and their cures. It is arranged in one alphabetical order, and embraces every disease that man, woman, or child is heir to. There are useful articles on chickenpox, diphtheria, measles, scarlatina, typhoid, etc., each from the pen of an expert in the treatment of the particular disease, and special articles on sanitation by Charles F. Wingate, sanitary engineer, New York City; snow-blindness by Dr. George S. Ryerson, of Toronto, Canada, and tropical diseases by Dr. Smith Ely Jelliffe, the chief editor. All branches of medicine and hygiene are described, and instructions are given for the nursing of the sick in the home.

In treating the subjects under discussion, the editors have avoided, wherever possible, the use of technical terms; this has been done without sacrificing scientific standards of accuracy, and in such manner that no less eminent an authority than Dr. William T. Bull, America's most eminent surgeon, has said "it impresses me as an admirable book which presents scientific facts to the laity, concisely, thoroughly, and accurately without furnishing information or uttering opinions which would encourage fads or quackery."

A useful chapter on poisons, their effects and antidotes, another on drugs and pharmaceutical preparations with indications of their uses in medicine, based upon the United States National Formulary, and a glossary of such technical terms as the editors found themselves compelled to use, together with an exhaustive index, complete the two handsome volumes into which this work has been compressed. It is embellished by twenty-four lithographed and full-page plates, and nearly 500 illustrations in black and white are interspersed thoughout the text.

This work is the first of its kind, the subject matter of which has been prepared by living medical writers whose ability to handle the topics entrusted to their care will be readily recognized by the educated men of the medical profession. For that reason, it is the first book on family medicine which has earned the endorsement of the medical profession.

Dr. Jelliffe holds a chair in the Columbia University, and is also associate editor of the *New York Medical Journal.*

---

Modern Medicine—Its Theory and Practice.—In Original Contributions by American and Foreign Authors. Edited by William Osler, M. D., Regius Professor of Medicine in Oxford University, England; formerly Professor of Medicine in Johns Hopkins University, Baltimore; in the University of Pennsylvania, Philadelphia, and in McGill University, Montreal. Assisted by Thomas McCrea, M. D., Associate Professor of Medicine and Clinical Therapeutics in Johns Hopkins University, Baltimore. In seven octavo volumes of about 1000 pages each. Illustrated. Volume II, just ready, Price per volume, cloth, $6.00, net; leather, $7.00 net; half Morocco, $7.50, net. Lea Brothers & Co., Publishers, Philadelphia and New York, 1907.

The early appearance of Volume II of this great work has a practical significance for the reader, quite apart from its indication of steady progress towards completion, for it ensures that the matter is fresh from the author's pen. The first volume dealt with General Medicine and the Diseases Caused by Physical, Chemical and Organic agents, and by Parasites of the Vegetable and Animal Kingdoms, and closed with chapters on Nutrition and that most important group of disorders which arise from faulty metabolism. The second volume disposes of one-half of the great modern class known as the Infections, which is to be finished in the third volume, due in the autumn. The fourth deals with Diseases of the Blood and Circulation, the fifth with those of the Alimentary Tract; the sixth with those of the Kidneys, of the Internally-secreting Glands, those of still obscure causation, and with Muscular, Vaso-Motor and Trophic Disorders. The seventh and last volume will cover Diseases of the Nervous System and of the Mind. This very brief résumé shows the simple classification, the judicious distribution of space, and the sustained interest of the work from beginning to end.

Reverting now to the contents of the volume in hand, Hektoen opens with an Introduction of the Study of Infections, following which McCrea considers Typhoid, Typhus, and Relapsing Fever; Councilman, Smallpox and Chickenpox; Dock, Vaccination; McCollom, Scarlet Fever and Diphtheria; Ruhräh, Measles; Rubella, the Fourth Disease, Erythema Infectiosum, Whooping Cough and

Mumps; Lord, Influenza; Coleman, Dengue; Koplik, Meningitis; Anders, Erysipelas; Musser and Norris, Pneumonia; Pearce, Toxemia, Septicemia and Pyemia; Poynton, Rheumatism; Dunbar, Cholera; Carroll, Yellow Fever; Calvert, Plague, and Shiga, Bacillary Dysentery.

Dr. Osler's position is such that the leaders of the profession of two Continents have stood ready to respond to his request to participate in the creation of this great work. His assignment of subjects shows his keen judgment of the men qualified to write with the highest authority. The resultant product will cover the whole field of medicine in its present advanced state of cultivation. It records the new level of knowledge which is open in its pages to every physician who desires the best information on any point of theory or practice.

---

THE PRACTITIONER'S LIBRARY OF GYNECOLOGY, OBSTETRICS AND PEDIATRICS.—In Original Contributions. By Eminent American and English Authors. THE PRACTICE OF GYNECOLOGY—Edited by J. Wesley Bovée, A. M., M. D., Professor of Clinical Gynecology in the George Washington University, Washington, D. C. Large octavo, 836 pages, with 382 engravings and 60 full-page plates in colors and monochrome. THE PRACTICE OF OBSTETRICS—Edited by Reuben Peterson, A. M., M. D., Professor of Obstetrics and Diseases of Women in the University of Michigan, Department of Medicine and Surgery, Ann Arbor, Mich. Large octavo, 1087 pages, with 523 engravings and 30 full-page plates in colors and monochrome. THE PRACTICE OF PEDIATRICS—Edited by Walter Lester Carr, M. D., Consulting Physician to the French Hospital; Visiting Physician Infants' and Children's Hospital, New York. Large octavo, 1014 pages, with 199 engravings and 32 full-page plates in colors and monochrome. Price per single volume, cloth, $6.00; leather, $7.00; half Morocco, $8.00. Price for any two volumes, cloth, $11; leather, $13; half Morocco, $15. Price for the three volumes, cloth, $15; leather, $18; half Morocco, $21.

Reviews of Peterson and of Carr have appeared in the JOURNAL. The publishers say:

"In preparing this new series the object has been to cover the whole domain composed of three cognate major specialties. Eminent American and English authors have united under the editorship of Drs. Bovee, of Washington; Carr, of New York, and Peterson, of Ann Arbor. By excluding those features of disease which are properly to be sought in works on general medicine, these volumes find space for a complete and comprehensive presentation of their respective subjects, with full practical details. Together with the most advanced knowledge of established value the authors have included their own observations, and the therapeutic measures which have resulted in the greatest success. This adds a personal element of obvious value. Abundant engravings and full-page plates illuminate the text, the facilities at command of the editors having enabled them to secure photographs and drawings exhibiting any point desired. Though it is manifestly to the advantage of every physician to have the whole library at hand, the volumes are sold separately for the convenience of those interested in the individual departments."

Dr. Bovée's work measures fully up to the standard, and may be taken as an exposition of the art and science of gynecology up to date. The full set will be a valuable and handsome addition to the physician's library, and it is meeting with a very large sale.

---

HARE'S THERAPEUTICS.—A Text-Book of Practical Therapeutics, with Especial Reference to the Application of Remedial Measures to Disease and Their Employment Upon a Rational Basis. By Hobart Amory Hare, M. D., B. Sc., Professor of Therapeutics and Materia Medica in the Jefferson Medical College of Philadelphia, Physician to the Jefferson Hospital, etc. New (12th) edition, enlarged and thoroughly revised to accord with the eighth decennial revision of the U. S. Pharmacopeia. In one octavo volume of 939 pages, with 114 engravings and four colored plates. Cloth, $4.00, net; leather, $5.00, net; half Morocco, $5.50, net. Lea Brothers & Co., Philadelphia and New York, 1907.

SCHLEIF'S MATERIA MEDICA AND THERAPEUTICS.—A Pocket Text-Book of Materia Medica, Therapeutics, Prescription Writing, Medical Latin and Medical Pharmacy. By William Schleif, Ph. G., M. D., University of Pennsylvania, Philadelphia. New (3d) edition, 12mo. 470 pages. Cloth, $2.50, net. Lea Brothers & Co., Philadelphia and New York, 1907.

The simultaneous appearance of two books on different phases of a large subject renders it advantageous to consider them together. "Schleif" is primarily a text-book on the fundamentals, devoting most of its space to Materia Medica, Pharmacology, Prescription Writing, Medical Latin, etc. "Hare" is equally a book for students and also for practitioners. It devotes one-half of its space to remedial agents, including both drugs and non-medicinal measures as well as foods, and the remaining half to diseases and their treatment, with copious and precise directions. Both parts are alphabetically arranged and fully cross-referenced, so that all information on any point can be quickly found. The "Index of Diseases and Remedies," with its suggestive annotations, is additionally helpful in practice. Dr. Hare's faculty for perceiving the pith of a matter and presenting it clearly is reflected in all his works and is the basis of their great popularity. For instance, this is the twelfth edition of his Therapeutics in seventeen years, and of most of the editions several large printings have been required. Thought it is the second edition since publication of the new Pharmacopeia, the author has not been content with a perfunctory revision, but he has, on the contrary, scrutinized every line and made changes and improvements wherever necessary to represent his very progressive subject.

Reverting for a moment to these two books for the purpose of considering them together from the student's standpoint, it may be said that they fit into each other very advantageously. Present-day curricula divide the subject very much on the lines here followed: a course in the pure Materia Medica and annexa in the first years, and then a course on the clinical applications, or Therapeutics proper. These two books contain more information than it is possible to put conveniently into a single volume. They divide it conformably to the best practice in teaching, and together they cover the whole subject.

PROGRESSIVE MEDICINE, Vol. II, June, 1907.—A Quarterly Digest of Advances, Discoveries and Improvements in the Medical and Surgical Sciences. Edited by Hobart Amory Hare, M. D., Professor of Therapeutics and Materia Medica in the Jefferson Medical College of Philadelphia. Octavo, 381 pages, with illustrations. Per annum, in four cloth-bound volumes, $9.00; in paper binding, $6.00; carriage paid to any address. Lea Brothers & Co., Publishers, Philadelphia and New York.

The June issue of Progressive Medicine (Volume II of the 1907 series) reviews many vitally important subjects, and embraces a wide range of interest. It opens with a thorough résumé of Hernia, by Dr. William B. Coley. The information therein is of value to every surgeon. Under the radical cure of femoral hernia many methods are discussed on their relative merits, hundreds of cases are summarized, and the after-results clearly stated. The best methods of procedure are freely illustrated. So also with inguinal hernia in children, umbilical hernia, cryptorchism, hernia through the foramen of Winslow, etc. In regard to the relative frequency of femoral to inguinal hernia, Dr. Coley writes:

"In a total of 1805 operations for hernia 130 were for femoral hernia."

---

THE PRACTICE OF OBSTETRICS.—By American Authors. Edited by Charles Jewett, M. D., Professor of Obstetrics in the Long Island College Hospital, Brooklyn, N. Y. In one handsome octavo volume of 786 pages, with 445 engravings in black and colors and 36 full-page colored plates. Cloth, $5.00, net; leather, $6.00, net; half Morocco, $6.50 net. Lea Brothers & Co., New York and Philadelphia.

It is quite a rare occurrence in medical literature to find a work of composite authorship appearing in successive new editions. Generally the demand finished by the first issue. This fact has been the subject of some speculation, the general explanation being that a composite book, perishing in what should be its youth, fails in adaptation to its environment in one or both of two essentials: it lacks either the homogeneity or the completeness of a good book by a single author. This defect, however, is individual with the book, and not inherent in the plan. There are, indeed, manifest advantages in handling a major subject by collaboration, the most conspicuous being the higher and more uniform level of authority thus attainable. Completeness without duplication is a matter of proper editing. Applying these considerations to a concrete case, it may be inferred that the demand for successive editions of Jewett's Practice of Obstetrics by American Authors indicates a well-edited work of proved high quality. Dr. Jewett and his corps of coauthors have again revised their work thoroughly to the latest date, both in text and in illustrations, in which it has always been notably rich. It is a work equally suited to the needs of obstetrician or student.

